# The Prognosis of Progressive Multifocal Leukoencephalopathy in HIV/AIDS Patients Undergoing Highly Active Antiretroviral Treatment: A Systematic Review

**DOI:** 10.7759/cureus.45155

**Published:** 2023-09-13

**Authors:** Soe Lwin Aye, Yash Trivedi, Zoryana Bolgarina, Heet N Desai, Mithum Senaratne, Shivling S Swami, Lubna Mohammed

**Affiliations:** 1 Internal Medicine, California Institute of Behavioral Neurosciences & Psychology, Fairfield, USA; 2 Obstetrics and Gynecology, California Institute of Behavioral Neurosciences & Psychology, Fairfield, USA

**Keywords:** neurological disorder of pml, treatment outcomes of hiv patients, highly active antiretroviral treatment, central nervous system disorder in hiv patients, prognosis of progressive multifocal leukoencephalopathy (pml)

## Abstract

Progressive multifocal leukoencephalopathy (PML), a viral infection of the central nervous system (CNS), is most commonly associated with advanced HIV infection. Although the severe neurological conditions - PML and progressive multifocal leukoencephalopathy immune reconstitution inflammatory syndrome (PML-IRIS) - are linked to HIV, little is known about their characteristics in the era of established antiretroviral therapy (ART).

The aim of this systematic review, which was performed by adhering to Preferred Reporting Items for Systematic Reviews and Meta-Analyses (PRISMA) 2020 guidelines, was to determine the prognosis of PML in patients with HIV. We gathered and examined articles, including case-control and cohort studies, systematic reviews, and meta-analyses that were published between January 1, 2013, and May 2023. These articles were compiled from the following databases: Pubmed, Pubmed Central, Google Scholar, Wiley Library, and ScienceDirect. A total of 519 records were found from these databases for our systematic review after applying the proper filters. They were then further screened and put through quality appraisal tools, which ultimately led to the selection of 10 articles for the final analysis. This research offers crucial insights into the clinical consequences of PML in HIV patients receiving highly active antiretroviral therapy (HAART).

## Introduction and background

Progressive multifocal leukoencephalopathy (PML), a viral infection of the central nervous system (CNS), is most frequently linked to advanced HIV infection [1]. Although PML and progressive multifocal leukoencephalopathy immune reconstitution inflammatory syndrome (PML-IRIS), two severe neurological conditions, are associated with HIV, little is known about their characteristics in the era of antiretroviral therapy (ART), which has been proven to be very effective [2]. PML, a rare but serious immune-mediated demyelinating disease of the CNS, is caused by human polyomavirus 2 or John Cunningham virus (JCV), which has a tropism for oligodendrocytes. Immunosuppressive therapies (for cancer, autoimmune illnesses, or organ transplants) and HIV infection have both been linked to the development of PML. Highly active antiretroviral therapy (HAART) may also occasionally worsen PML due to an autoimmune reaction stoked by the recovery of CD4+ T cell count, which results in PML-IRIS. This holds true even though HAART significantly lowers the incidence of PML. PML is characterized by demyelination, astrocyte changes, and aberrant oligodendroglial nuclei, while edema, lymphocyte infiltration, and blood-brain barrier disruption are frequently absent [3].

Patients often have localized neurologic impairments that progressively worsen when they first present with PML. Multiple symptoms, such as hemiparesis, aphasia, ataxia, visual impairments, and cognitive impairment, are typical in PML since it is a multifocal process [4]. Previously, HIV was the primary illness in around 80% of instances with PML; however, HAART has decreased the prevalence of HIV-PML and improved survival rates [5]. Diseases that cause severe systemic immunosuppression may cause JCV to get reactivated in the CNS, which could result in the growth of an active opportunistic JCV infection in CNS tissues. The exact series of actions that lead to this is unclear. Before the development of HAART for AIDS treatment (before 1996), AIDS was the most common cause of immunosuppression resulting in JCV-related CNS pathology, and PML was the most prevalent manifestation of JCV CNS involvement [6]. PML, which appeared in as many as 5% of HIV-positive individuals during the AIDS pandemic, is closely linked to HIV infection. HIV and JCV co-infection may have a synergistic effect that helps to explain this connection [7]. HAART is now regarded as the standard treatment for AIDS-related PML and has greatly increased patient survival in both AIDS and PML. The clinical condition known as immune reconstitution inflammatory syndrome is characterized by an overwhelming inflammatory response to a pre-existing antigen or pathogen and a paradoxical worsening of the clinical state following the start of antiretroviral medication [8-20]. IRIS appears in two ways: (1) ironically, when an already-diagnosed ailment gets worse after starting ART, and (2) getting unmasked when an undiagnosed condition is found after starting ART [1,7]. Since several pathogenic mechanisms contribute to the disease's manifestation, clinical results of PML might vary between patients, and this raises medical concerns in HIV/AIDS patients. The purpose of this systematic review is to gain deeper insights into the care plan of PML in HIV/AIDS patients.

## Review

Methods

Assessing the clinical outcomes of PML in HIV/AIDS patients undergoing HAART is the main objective of this study. The Preferred Reporting Items for Systematic Reviews and Meta-Analyses (PRISMA) 2020 guidelines were followed for conducting this systematic review.

Inclusion/Exclusion Criteria

We reviewed articles that were published between January 1, 2013, and May 2023 and included only those articles published in the English language. All the patients were adult men and women (aged above 18 years). Only articles with free full text available were selected. We reviewed case series, case reports, cohort studies, case-control studies, systematic reviews, literature reviews, and meta-analyses by using keywords such as "HIV/AIDS", "Progressive Multifocal Leukoencephalopathy", "HAART", "JC virus infection", "prognosis", and "treatment outcomes". We excluded articles published before 2013, studies involving animals, and articles published in other languages. Table 1 provides a summary of the keywords used, databases queried, and the total number of search results from each database.

**Table 1:** Keywords and search results PML: progressive multifocal leukoencephalopathy; JC virus: John Cunningham virus; HIV/AIDS: human immunodeficiency virus/acquired immune deficiency syndrome; HAART: highly active antiretroviral treatment

Keyword	Database	Filters applied	Search results
HIV infection OR AIDS OR ( "HIV Infections/drug therapy"[Majr] OR "HIV Infections/epidemiology"[Majr] OR "HIV Infections/immunology"[Majr] OR "HIV Infections/pathology"[Majr] OR "HIV Infections/therapy"[Majr] OR "HIV Infections/virology"[Majr] ) AND Highly active antiretroviral therapy OR Anti-HIV treatment OR ( "Antiretroviral Therapy, Highly Active/adverse effects"[Majr] OR "Antiretroviral Therapy, Highly Active/mortality"[Majr] ) AND Progressive Multifocal Leukoencephalopathy OR JC virus Infection OR ( "Leukoencephalopathy, Progressive Multifocal/classification"[Majr] OR "Leukoencephalopathy, Progressive Multifocal/complications"[Majr] OR "Leukoencephalopathy, Progressive Multifocal/drug therapy"[Majr] OR "Leukoencephalopathy, Progressive Multifocal/epidemiology"[Majr] OR "Leukoencephalopathy, Progressive Multifocal/etiology"[Majr] OR "Leukoencephalopathy, Progressive Multifocal/mortality"[Majr] OR "Leukoencephalopathy, Progressive Multifocal/pathology"[Majr] OR "Leukoencephalopathy, Progressive Multifocal/physiopathology"[Majr] OR "Leukoencephalopathy, Progressive Multifocal/virology"[Majr] )	PubMed	Free full text, age over 18 years, male and female, and 2013-2023	356
Prognosis of PML in HIV/AIDS AND JC virus infection in HIV/AIDS AND Treatment outcomes of PML in HIV/AIDS	Google Scholar	Any type of publications between 2013-2023	110
Progressive Multifocal Leukoencephalopathy AND Acquired Immune Deficiency Syndrome	ScienceDirect	2013-2023	25
Progressive Multifocal Leukoencephalopathy AND Acquired Immune Deficiency Syndrome	PubMed Central	2013-2023	26
Progressive Multifocal Leukoencephalopathy AND Acquired Immune Deficiency Syndrome	Wiley Online Library	2013-2023	2
		Total	519

Results

**Figure 1:**
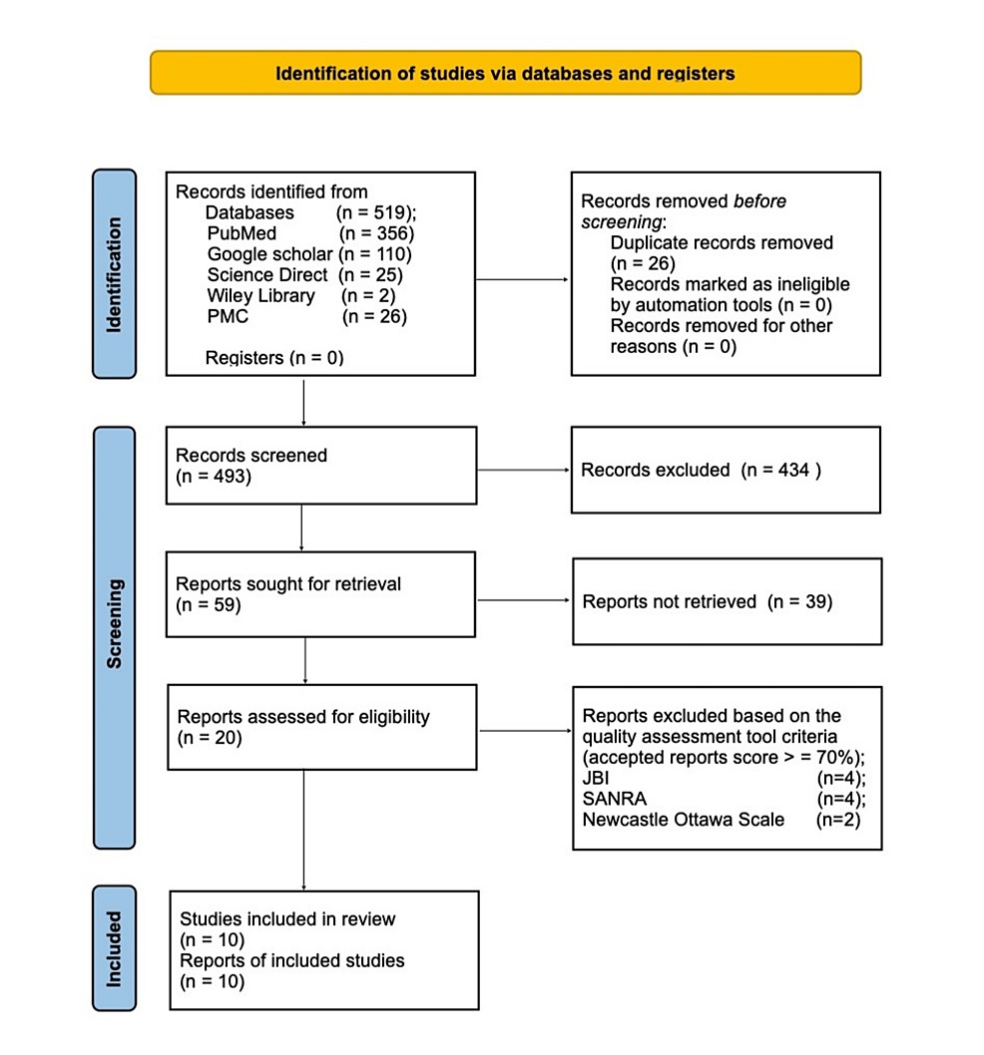
PRISMA flow chart PRISMA: Preferred Reporting Items for Systematic Reviews and Meta-Analyses; JBI: Joanna Briggs Institute; SANRA: Scale for the Quality Assessment of Narrative Review Articles

A search of the databases PubMed, Google Scholar, ScienceDirect, Wiley Online Library, and PubMed Central (PMC) by using the search technique and common keywords as well as applying filters like 2013 to 2023 yielded a total of 519 articles. The papers were then screened on the basis of the title and abstract. The articles were chosen based on their eligibility requirements and full-text accessibility. Only 10 publications were fully accessible, and they were all reviewed for quality, and a comprehensive PRISMA flow diagram was made. The studies included in the review underwent quality assessment using tools such as the Newcastle-Ottawa Scale for observational cohort studies, the Joanna Briggs Institute (JBI) checklist tool for case reports/series, and the Scale for the Quality Assessment of Narrative Review Articles (SANRA) checklist for literature review. An overview of the screening process is shown in the PRISMA flow chart in Figure 1.

The studies included in this systematic review are summarized below in Table 2.

**Table 2:** Characteristics of studies included in the systematic review PML: progressive multifocal leukoencephalopathy; HIV: human immunodeficiency virus; AIDS: acquired immune deficiency syndrome; IRIS: immune reconstitution inflammatory syndrome; JCV: human polyomavirus (or) John Cunningham virus; cART, combined antiretroviral therapy; HAART: highly active antiretroviral therapy

Sr. no	Author	No. of participants	Study type	Study subject	Conclusion
1	Arora et al., 2022 [1]	765	Retrospective cohort	PML and AIDS	Despite the implementation of HAART, PML is associated with considerable morbidity; nevertheless, mortality has significantly decreased if ART is initiated early. Early diagnosis and HAART are essential for positive outcomes
2	Summers et al., 2018 [2]	32	Case series	PML-IRIS and AIDS	With mortality rates above 50%, as shown in this study, and median survival of 266 days for patients with PML and 109 days for patients with PML-IRIS, PML and PML-IRIS continue to have poor prognoses in spite of the emergence of modern ART
3	Fanjul et al., 2013 [9]	98	Cohort	PML and AIDS	Lower mortality seen in the protease inhibitor group may be clinically important, and if so, a protease inhibitor-based therapy may be recommended for patients with PML
4	Shahani et al., 2015 [11]	1	Case report	PML-IRIS and AIDS	The first case of an HIV-positive patient successfully treated for PML IRIS with maraviroc. This case may have clinical implications as maraviroc can be used to augment the current HIV regimen and, at the same time, improve the outcome of PML-IRIS
5	Cambrea et al., 2017 [12]	2	Case report	PML and AIDS	In terms of prognosis, the location of lesions was more important than their size. For the patient who survived, cART with good CNS penetration and frequent physical therapy allowed for a partial recovery of motor deficiency, language, and communication
6	Crossley et al., 2016 [15]	2	Case report	PML and AIDS	Despite intermittently maintaining mild immunosuppression and HIV suppression, PML was initially remitted, but symptoms later developed and investigation findings were consistent with recurrent PML
7	Sharma et al., 2013 [17]	1465	Retrospective cohort	PML and AIDS	With a median survival of 7.6 months, advanced immunosuppression was frequently present upon diagnosis, and the overall prognosis was dismal. Patients who did not react to HAART had a quick course to mortality with significant morbidity linked to the development of neurological impairments
8	Badura et al., 2023 [18]	2	Case series	PML and AIDS	Patients with PML, regardless of the condition of their immune system, need and would benefit greatly from treatment that specifically targets JCPyV
9	Fournier et al., 2017 [19]	157	Retrospective cohort	PML-IRIS and AIDS	IRIS does not appear to affect AIDS-related PML results, despite the fact that it can be severe. This is evidence of a protective immune response that is important for JCV replication over the long term. The effectiveness of steroids cannot be definitively determined, although case reports indicate that early corticosteroid therapy may be suggested for PML-IRIS which is life-threatening
10	Pavlovic et al., 2015 [20]		Literature review	PML and AIDS	The incidence of PML in this cohort was significantly decreased by improvements in HIV infection therapy. The prognosis for HIV+ patients with PML is still bleak, with 50% of them passing away within the first two years of the commencement of the illness

Discussion

Pathophysiology of PML

JCV has been around for a long time and is very common. It appears to have coevolved with human populations. JCV infection primarily affects children through horizontal transmission during prolonged cohabitation, most frequently from parent to kid. It is believed that person-to-person contact, contaminated surfaces, food, and water can all contribute to the spread of the disease [5,7].

PML is a demyelinating disease of the CNS, which is a rare disease caused by the reactivation of JCV. Most patients with a primary infection that occurs in childhood do not show any symptoms [8]. The virus then remains dormant in the bone marrow, lymphoid, and renal tissues, but when cellular immunity is suppressed, the virus reactivates, travels to the CNS, and causes oligodendrocyte infection and demyelination.

It is characterized by subacute neurological degeneration, which frequently leads to deteriorating strength, sensory loss, dysarthria, ataxia, hemianopsia, and cognitive impairment [7-32]. It is unknown where specifically and how JCV acquired its neurotropism. The question of when and how JCV enters the CNS is still being debated. JCV has been found in the brains of healthy persons as well as those who have an immune system that is suppressed without PML. However, the significance of this reservoir in the brain is unclear [6,7]. Since PML only sometimes affects immunocompetent hosts, persistent cellular immunity deficiency is unquestionably a favorable environment for viral reactivation.

Histopathologically, the trio of larger, weird astrocytes with irregularly lobulated nuclei, multifocal demyelination, and oligodendroglia with enlarged hyperchromatic nuclei describes classic PML. In addition to the gray matter-white matter junction and the subcortical white matter, PML also affects the cortex and deep gray matter, which is significant. This likely explains why the disease's signature mix of cortical and subcortical symptoms appears to be caused by PML [7,22,23,32].

PML and IRIS

IRIS is characterized as a paradoxical deterioration of underlying infectious processes or the emergence of new symptoms after the start of HAART in HIV-infected patients. It frequently occurs after the quick immune function restoration and is self-limiting, but it can also be lethal or have long-term effects. There have been cases of deadly outcomes as a result of brain enlargement and herniation [8,26,30,31]. Clinical manifestations follow a continuum that is influenced mainly by the host's immunological response. At one end of the range is the absence of significant antiviral immune activity, which was common in the initial presentations of PML and which we refer to as "Classic PML”.

A gradient of presentations with a combination of clinical, imaging, and histological characteristics that show varying degrees of immune function preservation or that are the result of immunological reconstitution is present in the middle. We call these manifestations "Inflammatory PML." PML-IRIS, also known as extensive inflammation, is at the extreme of this gradient and can result in immune-mediated damage [7,23,26]. By inducing a rebound inflammatory response to previously undetected or asymptomatic infections, combined antiretroviral therapy cART-driven partial or full immune reconstitution can cause unusual presentations of classic opportunistic illnesses; originally known as "immune reconstitution syndrome," it was later referred to as "immune reconstitution inflammatory syndrome" (IRIS) [7].

IRIS is described as immune reconstitution accompanied by an increase in CD4+ T cell count or a drop in plasma HIV RNA levels, together with a dramatic exacerbation of neurological signs and symptoms. Based on the timing of the clinical manifestations, two types of IRIS presentations have been identified: unmasked IRIS, in which the inflammatory response to a subclinical infection causes it to become clinically manifest, and paradoxical IRIS, in which symptoms of a previously recognized infection worsen [7,8].

According to estimates, IRIS develops in 4-22% of people with HIV-related PML. IRIS can be life-threatening and associated with a mortality rate of 5-28%. Clinical signs are often minor, but if the concomitant inflammatory response is severe, which most frequently happens in patients with the steepest rise in CD4+ T cell counts, JCV-specific CD8+ and CD4+ T cells are detectable in the peripheral blood of survivors after effective immunological reconstitution. Notably, compared to typical PML, PML-IRIS is linked with fewer JCV-infected cells, indicating an effective reduction in infection. Inflammatory characteristics in PML are generally assumed to be related to increased survival rates even though some sources claim that this is not always the case [7,8,26,31]. Nevertheless, numerous investigations have been unable to show any appreciable variations in survival rates between PML with and without IRIS [5].

PML and HIV/AIDS

The epidemiology of PML was altered by the advent of AIDS. After its incidence increased by 50 times, HIV infection quickly became the main disease-predisposing factor [8,22,27]. As with HIV infection, JCV reactivation is most likely to occur in patients with low CD4+ T cell counts, and PML prognosis is correlated with CD4+ T cell levels. PML develops in various immunologically vulnerable patient populations as a late and uncommon symptom of JCV infection. In 1982, the first study on PML and AIDS was released. Although there was a startling increase in incidence, the clinical phenomenology of HIV-related PML was similar to what had previously been reported.

Prior to the development of cART, up to 8% of people with HIV were said to have PML, according to reports. Only 9% of patients survived longer than a year; the median survival was six months [7,8].

Classic PML manifests as the progressive deterioration of many neurological symptoms and might include supratentorial or infratentorial tissues. Expanding patches of demyelination that eventually consolidate result from lytic infection of oligodendrocytes. The symptoms vary depending on the sites involved; behavioral and cognitive disorders, sensory and motor impairments, ataxia, aphasia, and visual alterations in the cortex are among the most prevalent. Over the duration of the illness, 20-44% of patients experience seizures.

The introduction of cART in the mid-1990s, which enabled the functional reversal of immunodeficiency and decreased the overall incidence of PML among people with HIV, also altered the spectrum of clinical presentations of PML as pathological and radiological inflammatory features started to be described. It is significant to note that this alteration was linked to dramatic gains in survival in one year, which reached 60% [7]. The fact that more than half of long-term PML survivors have no or relatively mild disability is also significant [8,22].

Treatment Options for PML

With cART, the incidence dropped and the results got better. For HIV-positive individuals, immunological reconstitution with cART is advantageous and a successful PML treatment. Despite some encouraging case reports, no specific therapy has so far been demonstrated to be effective in small clinical trials [9,23,28]. A combination of medications that target several stages of the JCV life cycle appears to have a better result. The potential for passive and active immunity treatments and immunological competence "boosters" appears intriguing. Gene editing and other cutting-edge futuristic techniques are not that far off [10].

There are no particular antiviral medications available for JCV [5,10,11,23,28]. The widespread use of HAART in individuals with HIV infection was the cause of the rise in PML survival rates seen in recent years [17,22,23]. In a trial involving 98 Spanish PML patients, the use of antiretroviral regimens incorporating protease inhibitors was linked to a lower mortality rate [5,8,9,23].

IRIS is typically a temporary self-limited procedure, and hence HAART should not be stopped. Many groups have employed corticosteroids to control the inflammatory response and prevent a catastrophic worsening, but their use is a matter of debate because they can also intensify immunosuppression and impair the JCV-specific cellular immune response. Of note, 54 patients with PML-IRIS linked to AIDS who were treated with corticosteroids showed varying outcomes in a case series [23,25,26,28,31]. Another intriguing strategy is the use of a five-drug early-intensified antiretroviral combination to rapidly lower the HIV load and restore the JCV immune response [8]. It has been suggested that the CCR5 antagonist maraviroc be used to treat PML-IRIS, which has been clinically linked to the return of CCR5+ monocytes to peripheral blood after the disappearance of peripheral CCR7+ monocytes [11,19,23].

Nevertheless, finding efficient antiviral medicines continues to be the critical objective. JCV-targeting therapy is necessary and would be extremely helpful for patients with PML, regardless of the state of the patient's immune system [18]. Advances in gene therapy that enable the direct targeting of the viral genome could be extremely useful in this situation [7,23]. Last but not least, passive and active immunizations against JCV are feasible options currently under study. In one study, two patients who received the JCV-VP1 (viral capsid protein 1) vaccine and recombinant human interleukin 7 (IL-7) have also seen positive outcomes [23,28].

Outcomes

As with HIV infection, JCV reactivation is most likely in patients with low CD4+ T cell counts, and PML prognosis is correlated with CD4+ T cell levels [7,18]. Even though PML does not necessarily signal the latter stages of HIV infection, baseline CD4 cell counts of less than 100/ul are linked to a greater mortality rate [12]. Aside from HAART, there is no effective treatment for PML. The most efficient therapy strategy is the quick use of HAART in PML patients who are HIV-positive. It is undeniable that HAART has increased survival and marginally reduced the incidence of this infection. However, the prognosis for patients who develop PML is yet unknown. Today, the only approach to stop the formation of new instances of PML is to develop strategies to enhance early HIV diagnosis and treatment [5,9,11-14,17-19,22,28,30,32].

An increasing percentage of HIV-positive PML patients live through the illness but continue to have serious neurological abnormalities. Patients with PML must undergo extensive comprehensive rehabilitation therapy, but significant improvement is possible [9,14,22,24,25].

If cART is started early in the course of the disease, 44-83% of HIV-related PML survivors experience clinical stabilization or some improvement [7]. Even with a robust cART regimen, complete remission is not the norm. Although a full recovery is not anticipated, one-third of the patients may become well enough to be functionally independent [5]. It appears that disease progression is closely tied to the brain's affected regions [12,28]. Even though ARV is widely available, individuals with HIV-associated PML have mortality rates of up to 30% after a year and 50-60% after two years following the diagnosis [18-20].

Interestingly, in one large retrospective cohort study over 25 years between 1994 and 2019, it turned out that 33 of 45 HIV-infected patients (73%) with PML survived after one year of symptom onset. More impressively, 22% of study participants survived more than 10 years. There were five 10-year survivors who were diagnosed with PML-IRIS [22].

Although the likelihood of PML recurrence is uncertain, a small number of cases of recurrence in HIV-related PML have been documented years after the initial presentation and despite ongoing cART and HIV suppression. In two case reports, PML was initially found to be in remission but later developed symptoms and investigation results that were compatible with recurrent PML even when HIV suppression and moderate immunosuppression were intermittently maintained [7,15,27,28].

One study with two cases of PML reported that rehabilitation activities can improve the functional status and quality of life of patients with PML; both had severe right hemiplegia and motor aphasia. Approximately eight months following their initial presentation, they were ready for intensive multidisciplinary therapy. Their three-times-per-week outpatient rehabilitative daycare treatment, which lasted an average of seven months, led to a gradual but significant improvement in their functional status. Both patients were able to communicate, walk independently with the aid of an assistive device, and live at home after completing the rehabilitation program [16]. Patients showed improved functional outcomes after starting HAART in the context of interdisciplinary rehabilitation [24,25].

Limitations

This study has a few limitations. We only chose articles that were published between 2013 and 2023. As a result, there is a possibility that we may have missed some pertinent articles that were published earlier. Also, this evaluation did not include any studies that involved minors (i.e., those under the age of 18 years). Besides, this review included only free full-text articles published in English.

## Conclusions

Clinical outcomes of PML in HIV/AIDS patients represent a medical concern since they could vary among patients. The disease manifests on a spectrum of pathological processes. A proper diagnosis can enable medical interventions to extend life and rehabilitation activities to improve functional status and quality of life in PML. Despite the implementation of HAART, PML is still associated with considerable morbidity; nevertheless, mortality significantly decreases if ART is initiated early. Early diagnosis and HAART are essential for positive outcomes. Independent of the CD4 cell count, clinicians should have a low threshold of suspicion for a diagnosis of PML-IRIS in any HIV-infected patient experiencing unexplained neurological problems. In rare cases, the disease can progress very fast and lead to death within just weeks despite treatment being provided. However, intensive, multidisciplinary rehabilitation therapy can improve activities of daily living among those who survive PML-IRIS with neurological sequelae. Most importantly, there is no consensus on a specific treatment method other than HAART for PML-IRIS in AIDS patients, which is not promising in terms of achieving complete remission. The articles included in this study highlighted that there is an urgent need for clinical trials and further studies should be conducted. A few prospective treatment options are scientifically sound and still need to be clinically proven and evidence-based.

## References

[1] Arora S, Ahmad FM, Deshwal R, Behal P (2022). Study of clinical profile and outcomes in progressive multifocal leukoencephalopathy in acquired immunodeficiency syndrome patients in the highly active antiretroviral therapy era - an observational study. Indian J Sex Transm Dis AIDS.

[2] Summers NA, Kelley CF, Armstrong W, Marconi VC, Nguyen ML (2019). Not a disease of the past: a case series of progressive multifocal leukoencephalopathy in the established antiretroviral era. AIDS Res Hum Retroviruses.

[3] Naro A, Billeri L, Lauria P, Manuli A, Calabrò RS (2022). Toward improving functional recovery in AIDS-associated progressive multifocal leukoencephalopathy: a single case pilot study on a novel neuromodulation approach. Innov Clin Neurosci.

[4] Saylor D (2018). Neurologic complications of human immunodeficiency virus infection. Continuum (Minneap Minn).

[5] Kartau M, Sipilä JO, Auvinen E, Palomäki M, Verkkoniemi-Ahola A (2019). Progressive multifocal leukoencephalopathy: current insights. Degener Neurol Neuromuscul Dis.

[6] Harypursat V, Zhou Y, Tang S, Chen Y (2020). JC Polyomavirus, progressive multifocal leukoencephalopathy and immune reconstitution inflammatory syndrome: a review. AIDS Res Ther.

[7] Cortese I, Reich DS, Nath A (2021). Progressive multifocal leukoencephalopathy and the spectrum of JC virus-related disease. Nat Rev Neurol.

[8] Lima MA (2013). Progressive multifocal leukoencephalopathy: new concepts. Arq Neuropsiquiatr.

[9] Fanjul F, Riveiro-Barciela M, Gonzalez J (2013). Evaluation of progressive multifocal leukoencephalopathy treatments in a Spanish cohort of HIV-infected patients: do protease inhibitors improve survival regardless of central nervous system penetration-effectiveness (CPE) score?. HIV Med.

[10] Loignon M, Toma E (2016). Treatment options for progressive multifocal leukoencephalopathy in HIV-infected persons: current status and future directions. Expert Rev Anti Infect Ther.

[11] Shahani L, Shah M, Tavakoli-Tabasi S (2015). Immune reconstitution inflammatory syndrome in a patient with progressive multifocal leukoencephalopathy. BMJ Case Rep.

[12] Cambrea SC, Pascu C, Rugină S, Iancu AM (2017). Evolution of progressive multifocal leukoencephalopathy in HIV-infected patients. Two case reports. Med Arch.

[13] Falcó V (2013). Progressive multifocal leukoencephalopathy, a rare but devastating disease in AIDS patients. Indian J Med Res.

[14] Möhn N, Grote-Levi L, Hopfner F (2022). Innovative therapeutic concepts of progressive multifocal leukoencephalopathy. J Neurol.

[15] Crossley KM, Agnihotri S, Chaganti J (2016). Recurrence of progressive multifocal leukoencephalopathy despite immune recovery in two HIV seropositive individuals. J Neurovirol.

[16] Moreh E, Israel S, Korem M, Meiner Z (2017). Rehabilitation outcome of progressive multifocal leukoencephalopathy in HIV-positive patients: a report of two cases. Disabil Rehabil.

[17] Sharma SK, Soneja M, Ranjan S, Miglani S, Hari S, Sinha S, Wig N (2013). Progressive multifocal leucoencephalopathy in HIV/AIDS: Observational study from a tertiary care centre in northern India. Indian J Med Res.

[18] Badura B, Barczak S, Mikuła T, Wiercińska-Drapało A (2023). Rapid-progressing progressive multifocal leukoencephalopathy in two patients newly diagnosed with HIV: case series and review of literature. J Neurovirol.

[19] Fournier A, Martin-Blondel G, Lechapt-Zalcman E (2017). Immune reconstitution inflammatory syndrome unmasking or worsening AIDS-related progressive multifocal leukoencephalopathy: a literature review. Front Immunol.

[20] Pavlovic D, Patera AC, Nyberg F, Gerber M, Liu M (2015). Progressive multifocal leukoencephalopathy: current treatment options and future perspectives. Ther Adv Neurol Disord.

[21] Abrão CO, Silva LR, Souza LC, Bisso NM, Turchi MD, Guilarde AO (2020). AIDS-related progressive multifocal leukoencephalopathy. Rev Soc Bras Med Trop.

[22] Anand P, Hotan GC, Vogel A, Venna N, Mateen FJ (2019). Progressive multifocal leukoencephalopathy: a 25-year retrospective cohort study. Neurol Neuroimmunol Neuroinflamm.

[23] Iannetta M, Zingaropoli MA, D’Abramo A, Oliva A, Mastroianni CM, Vullo V, Rosa CM (2016). HIV-associated progressive multifocal leukoencephalopathy: current perspectives. Neurobehav HIV Med.

[24] Ryan M, Zack Mc, Leslie R (2013). A case of progressive multifocal leukoencephalopathy due to HIV/AIDS with functional improvement after acute inpatient rehabilitation. PM R.

[25] Nguyen BT, Gershkoff AM, Brown CH, Feng A (2016). Poster 346 Rehabilitation of progressive multifocal leukoencephalopathy: a case report. PM R.

[26] Corti M, Villafañe M, Trione N, Yampolsky C, Sevlever G (2013). Progressive multifocal leukoencephalopathy presenting as IRIS in an AIDS patient. A case report and literature review. Neuroradiol J.

[27] McEntire CR, Fong KT, Jia DT (2021). Central nervous system disease with JC virus infection in adults with congenital HIV. AIDS.

[28] Alstadhaug KB, Myhr KM, Rinaldo CH (2017). Progressive multifocal leukoencephalopathy. Tidsskr Nor Laegeforen.

[29] Singh TS, Singh K (2021). Acute cerebellar ataxia as the presenting symptom of progressive multifocal leukoencephalopathy with HIV - a case report. Neurol India.

[30] Chen YH, Chang JB, Wei JJ (2019). Three acquired immunodeficiency syndrome patients with central nervous system infection: diagnostic approach and outcome of treatment. Chin Med J (Engl).

[31] Hirsch HH, Kardas P, Kranz D, Leboeuf C (2013). The human JC polyomavirus (JCPyV): virological background and clinical implications. APMIS.

[32] Li J, Xue M, Yan S, Guan C, Xie R, Chen B (2021). A comparative study of multimodal magnetic resonance in the differential diagnosis of acquired immune deficiency syndrome related primary central nervous system lymphoma and infection. BMC Infect Dis.

